# Modulation of the Gut Microbiome to Improve Clinical Outcomes in Hepatocellular Carcinoma

**DOI:** 10.3390/cancers14092099

**Published:** 2022-04-23

**Authors:** Sj Shen, Saroj Khatiwada, Jason Behary, Rachel Kim, Amany Zekry

**Affiliations:** 1 Microbiome Research Centre, St George and Sutherland Clinical Campus, University of New South Wales, Sydney, NSW 2052, Australia; sj.shen@unsw.edu.au (S.S.); s.khatiwada@unsw.edu.au (S.K.); j.behary@unsw.edu.au (J.B.); rachel.kim3@student.unsw.edu.au (R.K.); 2 Department of Gastroenterology and Hepatology, St George Hospital, Sydney, NSW 2217, Australia

**Keywords:** microbiota, hepatocellular carcinoma, gut–liver axis, immune response, immunotherapy

## Abstract

**Simple Summary:**

Hepatocellular carcinoma (HCC), the most common form of liver cancer, is the third leading cause of cancer-related mortality worldwide. Recent research indicates that altering the gut environment affects liver immune response and cancer progression, through what is known as the ‘gut–liver axis’. HCC patients have dampened anticancer responses, but through modulating the gut environment, there is potential to reinvigorate these ‘exhausted’ immune cells to target tumors. In promising research on melanoma, transplanting stool from healthy donors through faecal microbiota transplantation (FMT) resulted in increased response to immunotherapy treatment in patients who were previously nonresponding. However, manipulating the gut environment as a therapeutic option in HCC remains to be explored. In this review, we explore the mechanisms through which this occurs, including how the gut environment affects gut barrier function, bacterial sensing, and liver immune responses, and how FMT may be a potential therapy for HCC patients nonresponsive to immunotherapy.

**Abstract:**

Hepatocellular carcinoma (HCC) is the third leading cause of cancer-related mortality worldwide. Recently, the gut microbiota has been shown to be closely linked to modulation of the immune and inflammatory responses, hence its potential as a therapeutic target. Although still under intense investigation, there exists a ‘gut–liver axis’ that links changes in the gut to the liver. In this regard, composition of gut microbiota and related metabolites, such as bile acids and short-chain fatty acids, have been shown to orchestrate key immune–metabolic events in liver disease and liver cancer. As hepatic immune cells are important determinants of antitumor responses, it is now increasingly recognized that the gut–liver axis plays a key role in influencing the intrahepatic immune response in HCC to favor a pro- or antitumor immune milieu. Hence, modulation of gut microbiota is potentially an attractive option to reinvigorate the antitumor responses. In this regard, promising evidence from melanoma preclinical and clinical studies has demonstrated the efficacy of gut-based intervention in reinvigorating the antitumor responses and improving responses to immunotherapy. However, the role of gut-based interventions as a therapeutic option in HCC remains to be elucidated. This review details how the gut microbiota and bacterial metabolites affect gut barrier function and ultimately immune response in HCC and raises the question of the potential of gut-based interventions as an adjunct therapy for patients with HCC receiving immunotherapy.

## 1. Introduction

Hepatocellular carcinoma (HCC) is the most common subtype of primary liver cancers, and the third leading cause of cancer-related mortality worldwide [1]. Patients with HCC often have underlying chronic liver disease, with major risk factors including excessive alcohol intake, hepatitis B or C virus (HBV/HCV) infection, obesity, and nonalcoholic fatty liver disease (NAFLD). Because of improved therapeutics for HBV/HCV-related HCC, NAFLD-related HCC is emerging as the most frequent cause of HCC in many countries [2,3]. However, treatment options for advanced HCC remain few and far between. Therefore, therapeutic strategies are needed to improve treatment outcomes in patients with advanced HCC.

Increasing evidence in cancer and inflammatory conditions indicate that the gut microbiota is key to the pathogenesis of many of these conditions and may be a target for immune modulation [4]. Gut dysbiosis is a term given to altered composition of the gut microbiota with loss of diversity and increased in abundance of particular species associated with disease. In chronic liver disease, several studies reported the presence of gut dysbiosis, and its association with the pathogenic process characterising the disease [5,6,7]. However, the mechanisms that link the gut and the liver in what is termed the ‘gut–liver axis’ are not well understood. Being anatomically linked via the portal vein, evidence is emerging that gut bacterial composition and related metabolites can travel to the liver and affect liver homeostasis. An understanding of the gut–liver axis and the mechanisms by which modulating the gut microbiota impacts hepatic inflammation and carcinogenesis will ultimately place manipulation of the gut microbiota as a potential therapeutic strategy. This review focuses on how gut bacteria, metabolites, intestinal barrier function, and immune responses link the gut–liver axis, and how ultimately modulating this gut–liver crosstalk may be a potential adjunct therapy for patients with HCC.

## 2. Gut Barrier Dysfunction and Bacterial Translocation

The gut barrier plays an integral role in preventing the translocation of bacteria from the gut lumen. A combination of factors, such as healthy epithelial cells, intact epithelial junctions, and mucous production, help to maintain epithelial integrity [8]. However, this process is disrupted during dysbiosis. Alterations to the gut microbiota disturbs gut homeostasis, including imbalance between commensals and pathogenic/proinflammatory bacteria, increases in bacterial ligands and enterotoxins, changes in nutritional balance and absorption, and mucin utilization [9]. These changes result in recruitment of immune cells and inflammatory events that disrupts epithelial integrity, which results in increased gut permeability and bacterial translocation, and subsequently potentiating the initial inflammatory responses [9,10,11]. Although we have evidence that dysbiosis precedes carcinogenesis in an animal model [12], it is difficult to know whether the same occurs in humans, or whether inherent inflammation and increased gut permeability causes dysbiosis. Nevertheless, emerging evidence implicates changes in the composition of the gut microbiota altering gut permeability and resulting in increased translocation of bacteria and bacterial components that induces hepatic injury. For instance, mice with a deficiency in toll-like receptor (TLR)-4 signalling were protected against diet-induced steatohepatitis [13], while in a rat model of steatohepatitis, antibiotics administration improved intestinal permeability, reduced TLR-4 and lipopolysaccharide (LPS) binding protein (LBP) expression in the liver and prevented fibrosis [14].

Gut barrier dysfunction plays a key role in the pathogenesis of HCC development. Therefore, serum levels of LPS have been shown to associate with the risk of HCC development [12,15]. In this regard, prolonged treatment of animal models of HCC with low-dose LPS, induced activation of TLR-4 signalling in resident liver cells, particularly hepatic stellate cells, with the activation of downstream mediators, led to generating a carcinogenic milieu. We have also reported that the progression of liver disease from inflammation to cirrhosis to liver cancer was associated with a gradient increase in serum LPS levels, which was further associated with a predominant intrahepatic immunosuppressive profile known to favour HCC development and progression [12]. Animal studies have proven that antibiotic treatment to deplete certain strains of gut bacteria can reduce tumour burden or prevent the development of HCC [16,17,18,19].

Results from preclinical models are supported by initial findings in clinical cohorts. The presence of gut-derived tight junction (TJ) proteins, such as zonula occludens (ZO)-1, and of bacterial endotoxins, such as LPS, in the plasma of patients with HCC indicates increased gut permeability [20,21]. Similarly, dysbiosis was associated with increased faecal calprotectin levels in patients with HCC [20]. These markers of disrupted gut barrier correlated with several proinflammatory markers such as high-sensitivity C-reactive Protein (hsCRP) and interleukin (IL)-5 [20,21]. Thus, increased gut permeability in patients with HCC appears to promote proinflammatory events, hence contributing to hepatic carcinogenesis. Therefore, there are robust data supporting the notion that gut barrier dysfunction and increased LPS production is among the key protumorigenic events in liver disease.

## 3. Gut Microbiota Derived Metabolites

Bile acids (BAs) form an integral part of the gut–liver axis. Produced in the liver, primary bile acids are secreted into the small intestine to facilitate digestion and absorption of lipids and certain vitamins [22]. Some of these primary BAs are then modified by gut bacteria to form secondary BAs, which are then partially reabsorbed [23,24]. The type of secondary BA formed has often been found to be dependent on the composition of gut microbiota, with some of the secondary BA significantly associated with liver pathologies [18,25].

Altered gut microbiota and bile acid signalling are observed in models of NAFLD and HCC. In germ-free animals or those depleted of gut microbiota through antibiotic treatment, total bile acid levels are significantly altered in different gastro-hepatic organs, serum, and faeces, indicating the importance of gut microbiota in the balance of BA signalling [24,26]. Feeding of cholic acid and other BA signalling intermediate molecules induced a global gene expression profile associated with HCC in a diet-induced mouse model of NAFLD [27]. In a separate study, cholic acid feeding in rats resulted in enrichment of Firmicutes (*Blautia* and *Allobaculum*) and a decrease in Bacteroidetes [28], which have been associated with HCC. These studies demonstrate the important bidirectional interactions between gut microbiota and BAs.

The role of gut microbiota and BAs were further demonstrated in mechanistic studies involving the administration of different BAs in animal models. Deoxycholic acid (DCA) as a secondary BA has been shown to promote hepatocarcinogenesis in obesity-induced HCC. Further, DCA production by modification of primary BAs in the gut correlated with the abundance of *Clostridium* spp. [29]. In a 7,12-dimethylbenz(a)anthracene (DMBA) and high fat diet-induced model of HCC, circulating DCA was shown to induce a senescence-associated secretory phenotype in hepatic stellate cells, leading to the production of proinflammatory and protumorigenic cytokines. Along this line, oral antibiotics treatment alleviated liver tumour burden, whilst concurrent addition of DCA increased tumour burden in the liver [29].

With respect to clinical cohorts, increased levels of primary BAs (cholic acid, chenodeoxycholic acid or total) and secondary BAs (ursodeoxycholic acid or total) were measured in sera of HCC patients compared to controls in the Singapore Chinese Health Study [30]. High levels of these BAs were noted to confer increased risk of HCC [30], paralleling animal studies. However, in a separate study, serum levels of 7-alpha-Hydroxy-4-Cholesten-3-one, an intermediate BA signalling molecule, were shown to be lower in nonalcoholic steatohepatitis (NASH) patients with HCC when compared to those without HCC [31]. The levels of total serum BAs, primary conjugated BAs, and intermediate molecules all correlated with an increased abundance of *Lactobacillus* strains [31]. In contrast to animal studies where excessive BAs are associated with HCC, the below-average levels of BAs in clinical studies also correlate with liver dysfunction, and this is reverted in patients who have undergone liver transplants [32]. Taken together, dysbiosis-induced dysregulation of BA signalling and the gut pool of BAs may contribute to dysregulated hepatic cytokine production and antitumour immune responses, thus leading to hepatic carcinogenesis, which may then feedback to promote dysbiosis and carcinogenesis (Table 1) [33].

Another group of metabolites of interest are short-chain fatty acids (SCFAs), which are produced through the microbial fermentation of dietary fibre. They are well known for their anti-inflammatory properties and has been shown to be protective in numerous animal models of allergic and inflammatory diseases [34]. However, the SCFA butyrate has been associated with obesity and shown to also induce insulin resistance and fat deposition in offspring rats [35].

The paradoxical capacities of SCFAs in inflammation and metabolic disorders are present in HCC. Initially in the context of metabolic syndrome, Singh et al. fed inulin to TLR5-deficient (T5KO) mice (which are prone to metabolic syndrome), and 40% of these mice showed lower body weight, fat pad, blood glucose, serum insulin, serum triglycerides, and other markers indicating amelioration of obesity, diabetes, and metabolic syndrome [36]. However, these same animals exhibited higher serum bilirubin, alanine transaminase (ALT), aspartate transaminase (AST), and alkaline phosphatase (ALP), all signalling liver dysfunction, in addition to significantly increased cecal butyrate levels [36]. Upon further investigation, these animals developed more aggressive liver disease and HCC [36]. Importantly, inulin feeding combined with a high fat diet induced early tumours in wildtype (WT) mice, and increased cancer penetrance in HCC-susceptible T5KO mice [36].

Since the HCC-susceptible T5KO mice also displayed dysbiosis and increased fibre-fermenting bacteria, it was shown that microbial transfer through cohousing was able to transfer susceptibility to HCC from T5KO to WT mice. Through administering butyrate for nine months, or by metronidazole-mediated depletion of butyrate-producing bacteria (and thus cecal butyrate levels), Singh et al. demonstrated that microbial fermentation of soluble fibre into butyrate was important in inducing HCC in susceptible mice. In addition, the same study also observed dysregulated bile acid metabolism [36], potentially due to the two pathways convergently relying upon the gut microbiota.

There have been few clinical studies that examined the association between SCFAs and HCC. Recently, we reported increased faecal and serum SCFAs in patients with NAFLD-HCC when compared to patients with NAFLD cirrhosis or non-NAFLD controls [37]. This was linked with enrichment of SCFA-producing bacteria, and increased bacterial genes involved in SCFA synthesis [37]. However, other studies suggested that SCFAs were reduced in patients with viral-related HCC [38], suggesting potential beneficial effects, albeit in HCC with a different aetiology.

In combination with aforementioned animal studies, it is imperative to delineate the protective and harmful effects of SCFAs in the development of HCC, especially when the protective effects of fibre are so widely publicised. Butyrate enhances intestinal barrier integrity and function to prevent bacterial and LPS translocation [39], which aids in the prevention of inflammation in HCC; however, SCFAs also enhances regulatory T cell (Treg) functions and shift towards immunosuppressive immune responses and cytokine milieu [40] that creates an environment for the initiation and potentiation of tumour growth. Recently, serum SCFA levels were associated with poorer anticytotoxic T lymphocyte antigen 4 (αCTLA4) response in patients with metastatic melanoma, which paralleled their findings in a mouse model whereby administration of butyrate in mice reduced αCTLA4 efficacy [41]. Overall, the role of SCFAs in cancer remains an interesting area of further research (Table 1) [42].

In addition to BAs and SCFAs, many other metabolites are being associated with liver inflammation. Bacterial metabolites from tryptophan metabolism have been shown to activate aryl hydrocarbon receptors, affecting the response to immunotherapy. Mice fed with high fat and high cholesterol diet developed NASH-HCC spontaneously, with a reduction in gut bacteria associated with tryptophan-metabolizing capacity and consequently lower levels of serum 3-indolepropionic acid when compared to control mice [43]. In line with this, in a clinical study, we found elevated faecal kynurenine and kynurenic acid and lower tryptophan and indole-3-carboxylate levels in patients with NAFLD-HCC when compared to NAFLD-cirrhosis [37], suggesting the role of indoles in HCC pathogenesis. In the search for other metabolites associated with HCC, a recent integrated metabolomic analysis of the systemic circulation, portal vein, tumour tissue, and stool samples of HCC patients found significantly different metabolite profiles to healthy controls [44]. Metabolites that were elevated in the portal vein or tumour tissue were associated with impairment of liver function, while linoleic acid and phenol (metabolites that were depleted in the portal vein and stool samples of HCC patients) were able to inhibit the growth of HCC cell lines in vitro [44]. Undoubtedly, further research aimed at modifying the gut microbiota and metabolite profile to one that is favourable to HCC (Table 1) may hold important therapeutic applications.

**Table 1:** Preclinical and clinical associations between metabolites and HCC.

	Study Design	Observations	Associations with HCC	Ref.
*Preclinical*				
CA feeding	C57BL/6 DEN model Chow, HFD + cholesterol ± CA (HFHCCA)	↗ Hepatic cholesterol ≈ Mitochondrial dysfunction genes	≈ NAFLD/HCC	[27]
	WKAH/HkmSlc rats Chow, +1.25 mmol/kg CA (M-CA), +5 mmol/kg CA (H-CA)	**(+ CA)** ↗ Firmicutes (*Blautia* and *Allobaculum*) ↘ Bacteroidetes **(H-CA vs. chow)** ↗ Faecal CA, αMCA, DCA, TCA, TDCA, 7-oxo-DCA ↘ Caecal total SCFAs, acetate, butyrate		[28]
DCA feeding	C57BL/6 DMBA + HFD; Abx ± DCA	**(HFD + Abx)** ↘ Tumour, *Clostridium* spp., serum DCA **(HFD + Abx + DCA)** ↗ Tumour, *Clostridium* spp., serum DCA	↗ HCC	[29]
Inulin feeding	C57BL/6, T5KO mice HFD + inulin	**(40% of mice)** ↘ Metabolic syndrome ↗ serum bilirubin, ALT, AST, ALP ↗ Serum total BA, unconjugated and conjugated: CA, αMCA, βMCA, ωMCA, CDCA, DCA ↗ Cecal butyrate ↗ Serum immunoreactivity: LPS, flagellin	↗ Liver disease and HCC ≈ Dysregulated bile acid metabolism ≈ Dysbiosis	[36]
	C57BL/6, T5KO mice HFD + inulin; CoH; Cf; GF	**(CoH, Cf)** ↗ Tumour, serum AFP, ALT **(GF)** ↘ Tumour, serum AFP, ALT	Gut microbiota transfers risk of HCC	
	C57BL/6, T5KO mice HFD + inulin; Abx	↘ Butyrate-producing bacteria ↘ Caecal butyrate, ↗ Serum AFP, ALT	↘ HCC	
Tryptophan	C57BL/6 HFD + high/low cholesterol (HC/LC)	**(HFD + HC diet)** Spontaneous HCC ↗ Serum and hepatic cholesterol ↘ gut bacteria associated ≈ tryptophan metabolizing capacity ↘ Serum 3-indolepropionic acid	↗ HCC	[43]
*Clinical*				
BA signalling	Singapore Chinese Health Study Sera of HCC patients	↗ 1° BAs (Total, CA species, CDCA species) ↗ 2° BAs (Total, UDCA species) ↘ 2°:1° BA ratio	↗ Risk of HCC	[30]
	Sera of HCC patients	↘ Serum 7-α-Hydroxy-4-Cholesten-3-one	≈ HCC with cirrhosis	[31]
SCFAs, others	Serum, liver, and stool samples from healthy, NAFLD-cirrhosis, and NAFLD-HCC patients, or healthy and HCC in vitro	↗ Faecal oxaloacetate, acetate, butyrate, formate, kynurenine and kynurenic acid ↘ Faecal tryptophan and indole-3-carboxylate ↗ Serum butyrate, propionate, malonate	≈ HCC	[37,44]
		Linoleic acid and phenol	↘ HCC cell line growth in vitro	[44]

↗ increases/promotes; ↘ decreases/alleviates; ≈ associated with; (bolded text): experimental intervention examined. T5KO, TLR-5 deficient; Abx, antibiotics; CoH, cohousing; Cf, cross-fostering; DMBA, 7,12-dimethylbenz(a)anthracene; GF, germ-free; HC, high cholesterol; HFD, high fat diet; 1°, primary; 2°, secondary; BA, bile acid; CA, cholic acid; CDCA, chenodeoxycholic acid; DCA, deoxycholic acid; MCA, muricholic acid; TCA, tauro-cholic acid; TDCA, tauro-deoxycholic acid; UDCA, ursodeoxycholic acid; SCFA, short-chain fatty acid.

## 4. Gut/Liver Immune Response

The current consensus in the context of HCC is that chronic cycles of inflammation and tissue repair eventuate in a switch to increased immune tolerance (increased anergic dendritic cells (DCs) and regulatory T cells (Tregs)), immunosuppression of antitumour immune cells (CD8^+^ T cells, natural killer (NK), natural killer T (NKT) cells, and innate lymphoid cells (ILCs)), and increases in protumorigenic leukocytes (tumour-associated macrophages (TAMs), tumour-associated neutrophils (TANs), and myeloid-derived suppressor cells (MDSCs)), which has been the focus of recent studies and reviews [45,46,47,48].

**Figure 1:**
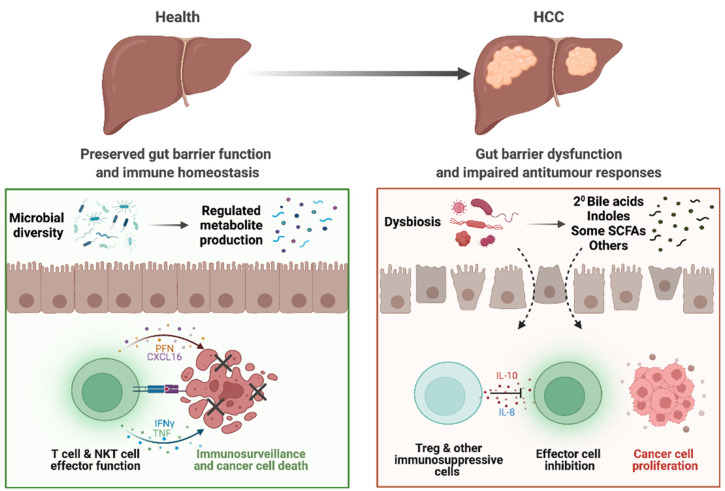
Altered gut–liver axis in patients with HCC. In health, the gut microbiota and bacterial metabolites produced maintain homeostasis of the gut. Preserved gut barrier functions prevent bacterial translocation and subsequent inflammation and allows only selected metabolites through into the circulation. Ultimately, homeostasis in the liver is also maintained, with immune surveillance that prevent hepatocarcinogenesis. However, in patients with HCC, dysbiosis results in dysregulated metabolite pool, including increased bile acids (BAs) and dysregulated BA and short-chain fatty acid (SCFA) signalling. There is also impaired gut barrier function, allowing for translocation of bacteria, bacterial components, and metabolites that induces inflammation. In the liver, these factors also play a role in impairing CD8+ T cell antitumour effector functions and increases Treg functions and immunosuppression. Together, these tumorigenic responses may contribute to the initiation and potentiation of HCC.

Dysbiosis-associated changes in metabolite profiles, gut barrier impairment, and bacterial translocation ultimately affect the homeostasis of hepatic immune response and induce carcinogenesis (Figure 1). We and others have shown dysbiosis and dysregulated immune responses (especially in T cell subpopulations), are associated with HCC disease in patients and animal models [12,38,49]. In viral-related HCC, patients with high levels of CD8^+^ T cells that are reactive to commensal gut bacteria *Bifidobacteria longum* and *Enterococcus hirae* showed a longer disease-free period [50], further highlighting the importance of gut microbiota in modulating antitumour T cell responses during HCC.

Clinical studies also indicate microbiota-mediated immune responses in HCC. *Bacteroides* were most represented in HCC patients and accompanies peripheral immune responses involving IL-8, IL-13, activated circulating macrophages (CD14^+^PDL1^+^, CD14^+^DR^+^PDL1^+^) and monocytic MDSCs (CD14^+^DR^low/neg^PD1^+^) [20]. In contrast, *Akkermansia* was represented mostly in healthy individuals, and *Bifidobacteria* in cirrhotic patients without HCC, and both genera were inversely associated with gut permeability (faecal calprotectin) and peripheral immune responses, including circulating activated circulating macrophages, monocytic MDSCs, chemokines CCL-3, -4, and -5, and cytokines IL-8 and IL-13 [20]. HCC-associated dysbiosis and increased immunosuppressive profiles were also observed in our study, with NAFLD-HCC patients showing increased proportions of peripheral blood Tregs and reduced CD8^+^ T cells [37]. These indicate that NAFLD-HCC may be associated with changes in gut bacterial composition, which in turn promote an immunosuppressive peripheral immune profile that contributes to HCC development and progress.

Data pertaining to the effect of the gut microbiota on the immune response are relevant for several reasons. There is evidence from clinical studies that increased Tregs [49] and decreased CD3^+^ and CD8^+^ T cells [51,52] in the peripheral blood and tumour samples of HCC patients carries worse prognosis as it is associated with reduced relapse-free survival [52]. This is supported in a diet-induced mouse model of HCC whereby systemic depletion of Tregs using diphtheria toxin in FoxP3-DTR mice (mice with diphtheria toxin receptor on FoxP3+ cells) prevented the progression of NASH to HCC [53].

Relevantly, modulation of the gut microbiota and its related metabolite profile has the potential to reinvigorate antitumour responses. Thus, inhibition of BAs through antibiotics (inhibiting bacterial conversion to secondary bile acids) or cholestyramine (sequestering bile acids) resulted in upregulation of primary bile acids and recruitment of NKT cells that in turn killed tumour cells through CD1d-dependant mechanisms [18]. This was through BA signalling to liver sinusoidal endothelial cells to produce CXCL16, which recruited natural killer T (NKT) cells to exert antitumor surveillance and was able to suppress the growth of both primary and metastatic cancer [18]. In line with this, activated NKT cells secrete both type 1 and 2 cytokines [54], including interferon (IFN)-γ and tumour necrosis factor (TNF), which are important in protection against metabolic disease and have roles in antitumour responses [55]. Further research investigating the exact mechanisms that link gut microbiota, metabolites, and antitumour responses in HCC will enable the gut microbiota to be a prime therapeutic target [56].

## 5. Targeting the Gut Microbiota for Improved Immunotherapy Outcomes

Immunotherapy harnesses the host immune system for antitumour effects. We have come a long way from the early interventions involving bacterial inoculation to activate the immune system in fighting cancers [57]. Our deeper understanding of the immune cells and markers associated with cancers has been crucial in advances in immunotherapy [58]. Current immunotherapies include immune checkpoint inhibitors (ICIs) that target immune checkpoint molecules (including programmed cell death protein 1 (PD-1) and CTLA-4) on T cells. In HCC, increased expression of PD-1 and its ligand programmed death-ligand 1 (PD-L1) have been correlated with tumour aggressiveness and recurrence following resection [59,60]. In patients with advanced stage HCC, kinase inhibitors have been the mainstay treatment since 2007 [61]. Recent progress in the development of ICIs has seen its use as a monotherapy, combination therapy with other ICIs, combination therapy with kinase inhibitors or vascular endothelial growth factor (VEGF) inhibitor [62].

Relevantly, the gut microbiota has been shown to influence response to ICIs. For instance, antibiotics use shortly before, during, or after treatment with αPD1/PD-L1 monoclonal antibodies resulted in significantly lower progression-free survival and overall survival in patients with epithelial cancers (nonsmall cell lung cancer (NSCLC), renal cell carcinoma (RCC) and urothelial cancer) when compared to those who had not received antibiotics [63]. Human nonresponders exhibited a low abundance of *Akkermansia muciniphila* in the gut microbiota, with supplementation of this bacteria improving response to immunotherapy [63]. This was supported by parallel studies in mice inoculated subcutaneously with either RET melanoma cells or MCA-205 fibrosarcoma cells, with antibiotics administration having a negative impact on the response to αPD-1 alone or in combination with αCTLA-4 [63].

With these promising findings indicating the modulatory role of the gut microbiota on response to ICIs, the group assessed the efficacy of faecal microbiota transplant (FMT) in cancer immunotherapy. Antibiotics-pretreated mice or germfree mice were orally administered with stool samples from donor patients with NSCLC or RCC, inoculated with MCA-205 cells, and subsequently treated with αPD-1 [63]. Interestingly, FMT from donors who were nonresponders to immunotherapy resulted in recipient mice not responding to αPD-1 treatment [63]. When mice received FMT from donor responders, they exhibited decreased tumour size, increased accumulation of CD4^+^CXCR3^+^ tumour infiltrating lymphocytes (TILs), and increased expression of PD-L1 on splenic CD4^+^ cells [63]. Further experiments elucidated the capacity of *A. muciniphila* to increase CD4^+^CXCR9^+^ TILs and CXCR9 expression on central memory T cells in the mesenteric lymph node, to reduce the number of tumour-infiltrating Tregs, and to stimulate the production of IL-12 [63]. This modulation towards increased antitumour responses likely explains the ability of *A. muciniphila* to reduce tumour size in vitro and to induce sensitivity to ICIs in vivo in mice that received FMT from nonresponders [63]. This extensive study establishes in animal experiments the capacity of the commensal gut bacteria to modulate antitumour immune responses and transfer sensitivity to immunotherapy through FMT.

Importantly, these preclinical findings established the way for human studies whereby patients with metastatic melanoma, refractory to ICI therapy, received FMT from responders to ICI. In a phase I clinical trial by Baruch et al., FMT from one donor was administered to the donor and four other participants, and similarly for stool from a second donor, and then the participants were reinducted with anti-PD-L1 therapy [64]. Out of the 10 participants, 3 out of 10 participants demonstrated clinical responses and tumour regression following FMT, with only mild adverse events being reported [64]. In this study, all three participants that responded to immunotherapy following FMT received samples from the same donor [64], indicating the choice of donor stool may be critical in inducing sensitivity to immunotherapy. In participants that responded, there is increased gut infiltration of antigen presenting cells, and increased TILs including DCs and CD8^+^ T cells [64]. Although all participants had increased abundance of bacteria associated with favourable outcomes of immunotherapy, the authors noted that this pilot trial is statistically powered for testing of safety only [64].

Similarly, in a back-to-back phase I study, Davar et al. also found that human nonresponders to ICIs showed increased sensitivity to subsequent immunotherapy following FMT from donor responders [65]. Single-donor-derived FMT was administered from seven donors (with either complete or partial response to ICI) to 16 nonresponder recipients, along with pembrolizumab [65]. Out of the 15 recipients able to be evaluated for response, three subjects showed objective responses, and another three subjects showed stable disease up to 18 months following FMT [65]. Interestingly, the three responders received FMT from the same donor [65], again indicating certain gut microbiota composition may be better at transferring sensitivity to immunotherapy. However, another recipient also received FMT from this same donor [65], but showed progressive disease after 3 months following FMT, suggesting host and other factors may contribute to determining the efficacy of FMT on response to ICI.

Further investigations indicated that all responder recipients showed a shift in gut microbiota composition towards that of the donor, with enrichment of Firmicutes and Actinobacteria, and a decrease in Bacteroidetes [65]. Responder recipients also showed increased CD56^+^CD8^+^ T cells (CD56 being a marker of enhanced cytotoxic functions) in the peripheral blood and increased gene expression of human leukocyte antigen (HLA) Class II genes, CD74, and granzyme K (GZMK), suggesting activation of circulating and tumour-infiltrating T cells [65]. There were also reductions in CCL2, IL-8, and IL-18, which had been correlated with negative outcomes to αPD-1 therapy [65]. In nonresponders, there were higher numbers of myeloid cells and Tregs in the tumour, with myeloid cells having significantly higher gene expression of IL-8 [65], these observations characteristic of tumour microenvironments. These two human FMT studies in melanoma demonstrated that FMT with subsequent αPD-1 therapy is not only safe, but also shows promise in altering the gut microbiota, metabolite profiles, and, ultimately, skewing towards an antitumour immune response in initially ICI nonresponders [64,65]. Thus, FMT as an adjunct therapy to immunotherapy is a promising strategy for a subset of patients in improving treatment outcomes.

## 6. Conclusions

The gut–liver axis is emerging to be an important link in research on HCC. The mechanism through which dysbiosis may contribute to liver cancer could be a series of physical, chemical, and physiological alterations, including impairment of gut barrier function, altering the gut and serum metabolite profile, and potentiation of cancer-associated immune responses. Encouragingly, preliminary studies have reported that gut microbiota can be modulated to reinvigorate the host anticancer immune responses. Ultimately, modulation of the gut microbiota could become an important adjunct therapy for advanced stage of HCC or to enhance response to immunotherapy in HCC.
